# Efficacy and safety of acupuncture for functional dyspepsia: an updated meta-analysis of randomized controlled trials

**DOI:** 10.3389/fmed.2026.1718632

**Published:** 2026-02-09

**Authors:** Xing-Xian Li, Zhuo-Ya Hu, Zi-Chen Li, Juan Tang, Yun-Yu Liu, Dong-Hua Zeng, Qian-Qian Zhou, Wen-Bin Ma, Lei Lan, Li Wang

**Affiliations:** 1 Acupuncture and Tuina College, Chengdu University of Traditional Chinese Medicine, Chengdu, China; 2 School of Health and Rehabilitation, Chengdu University of Traditional Chinese Medicine, Chengdu, China; 3 Department of Anaesthesia, McMaster University, Hamilton, ON, Canada; 4 Michael G. DeGroote Institute for Pain Research and Care, McMaster University, Hamilton, ON, Canada; 5 Department of Health Research Methods, Evidence and Impact, McMaster University, Hamilton, ON, Canada

**Keywords:** acupuncture, functional dyspepsia, meta-analysis, randomized controlled trial, systematic review

## Abstract

**Aim:**

Acupuncture has been used for the treatment of functional dyspepsia (FD); however, its effects remain uncertain. We aimed to assess the efficacy and safety of acupuncture for FD using systematic review and meta-analysis of randomized trials (RCTs).

**Methods:**

We searched six databases and two trial registries up to March 13, 2025. Paired reviewers screened literature, extracted data, and assessed the risk of bias. We performed meta-analyses using random-effects models and assessed the certainty of the evidence using GRADE approach.

**Results:**

We included 23 RCTs (2,454 participants). Compared to sham acupuncture, high to moderate certainty evidence shows that acupuncture probably improves FD symptoms (weighted mean difference [WMD] −14.46 points on the 195-point NDSI, 95% CI −16.31 to −12.62) and quality of life (WMD 10.39 points on the 100-point NDLQI, 95% CI 7.06 to 13.73) without an increase in adverse events (relative risk 1.15, 95% CI 0.63, 2.09). Compared to no treatment or usual care, moderate certainty evidence shows that acupuncture probably improves FD symptoms (WMD −20.19 points on the 195-point NDSI, 95% CI −30.22 to −10.15). When compared with prokinetics (itopride, mosapride, and domperidone), acupuncture probably improves quality of life (WMD 5.69 points on the 100-point NDLQI, 95% CI 4.36 to 7.02) and may improve FD symptoms (WMD −17.40 points on the 195-point NDSI, 95% CI −29.08 to −5.72).

**Conclusion:**

Acupuncture probably improves FD symptoms and quality of life when compared with sham acupuncture, no treatment or usual care, and prokinetics.

**Systematic review registration:**

https://doi.org/10.1002/14651858.CD008487

## 1 Introduction

Functional dyspepsia (FD) is a common but under-recognized syndrome characterized by bothersome and recurrent postprandial fullness, early satiety, or epigastric pain/burning, but without any structural abnormalities detected via imaging or endoscopy. A meta-analysis of 100 population-based studies comprising over 312,000 participants found that the pooled prevalence of uninvestigated dyspepsia was 21% with a 95% confidence interval (CI) [18, 24%] (1). These symptoms are attributed to impaired gastric motility, visceral hypersensitivity, and inflammation of gastric and duodenal mucosa. In addition, psychiatric comorbidities—including psychiatric comorbidities and specific personality traits—are increasingly recognized as contributors to FD pathophysiology.

FD Management primarily focuses on symptom relief, with treatment options including proton pump inhibitors, H2-receptor antagonists, prokinetic agents, and antidepressants (2). However, many patients experience only partial relief, face recurring symptoms, and suffer adverse drug effects, which significantly impact the quality of life (3). This suggests that pharmacological therapy, the most frequently used one, is insufficient to relieve FD patients. These challenges have prompted growing interest in complementary therapies, such as acupuncture.

Acupuncture, a core therapeutic modality in Traditional Chinese Medicine, involves stimulating specific anatomic points (‘acupoints’) using fine needles to elicit therapeutic effects (4). Now practiced in more than 180 countries, acupuncture encompasses diverse techniques with regional features, including Traditional Chinese acupuncture, dry needling, and others (5). For dyspepsia management, manual acupuncture has traditionally been favored, though electroacupuncture—where electrical stimulation is applied through connecting the needles—has gained popularity in recent decades (6).

FD is increasingly understood as a disorder of gut-brain interaction, with gastrointestinal dysmotility and central sensitization as central features (7, 8). More than half of FD patients experience sleep problems and mental disorders (9), and long-term cohort data suggest a strong association between depressive symptoms and FD onset (10). Acupuncture has been shown to improve gastric emptying, accommodation (11, 12), and functional brain connectivity, thus potentially restoring balance along the brain-gut axis (13–15).

Previous systematic reviews assessed the effects of acupuncture for FD (16–26); however, they present several important limitations, including outdated literature searches; inconsistent diagnostic criteria for FD; reliance on standardized mean difference (SMD) as the effect measure, which is difficult to interpret; use of fixed-effects model despite evident clinical and methodological heterogeneity; lack of subgroup analysis to explore sources of heterogeneity; and failure to assess the overall quality or certainty of evidence.

To address the limitations, we aimed to systematically assesses the efficacy and safety of acupuncture in the treatment of FD.

## 2 Methods

Our systematic review followed the Preferred Reporting Items for Systematic Reviews and Meta-Analyses (PRISMA) statement (27). The review protocol was registered with the Cochrane Database of Systematic Reviews,^1^ and we updated our previous Cochrane review first published in 2014 (26). Prior to literature screening, we modified the inclusion criteria of our protocol by restricting the eligible randomized controlled trials (RCTs) that must follow up FD patients for at least 4 weeks.

### 2.1 Literature search

We searched the following electronic literature databases from the inception to March 13, 2025, including PubMed, Embase, Cochrane Library, Chinese National Knowledge Infrastructure (CNKI), VIP Database, and Wanfang Database (Supplementary Table 1). We also searched ClinicalTrials.gov and the WHO International Clinical Trials Registry Platform (ICTRP). Additionally, using the updated eligibility criteria, we screened the trials that were included, excluded, or awaiting assessment in our previous Cochrane review published in 2014 (26) and the reference lists of other related systematic reviews.

### 2.2 Study selection

We included RCTs that (1) enrolled adult patients (≥18 years of age) diagnosed with FD based on the Rome II, III, or IV criteria, without restrictions regarding gender or race; (2) randomly assigned participants to either an acupuncture group (manual acupuncture or electroacupuncture) or a control group (sham acupuncture, no treatment, or standard pharmacological intervention); (3) follow them for at least 4 weeks; and (4) reported at least one of the following outcomes: primary outcomes: symptom relief and quality of life; secondary outcomes: mental health (anxiety and/or depression) and adverse events (any reported adverse event). We excluded studies that (1) involved participants with structural, systemic, or metabolic diseases, severe psychiatric sickness, or a history of abdominal operations; (2) compared different acupuncture techniques or acupuncture with Chinese herbs, or used different co-interventions in the treatment and control groups; and (3) were semi-RCTs.

Two pairs of reviewers (Zi-Chen Li, Xing-Xian Li, Qian-Qian Zhou, and Zhuo-Ya Hu) screened titles, abstracts, and full texts, independently and in duplicate, according to predefined instructions. Disagreements were resolved through discussion or adjudication by the third reviewer (Lei Lan).

### 2.3 Data abstraction and risk of bias assessment

Two reviewers (Zi-chen Li and Dong-hua Zeng) extracted data using standardized, pilot-tested forms independently and in duplicate, including studies characteristics (trial design, funding, country), participants (age, sex, and duration of the condition), interventions (details of acupuncture and control, frequency and duration of treatment, and co-interventions), and outcomes of interest listed above. Disagreements were resolved through discussion or adjudicated by the third reviewer (Lei Lan). When a study reported results at multiple time points, we selected data from the time-point most commonly reported—4 weeks—or the time point closest to 4 weeks. We calculated change data using both baseline and the end-of-study data and its correlation coefficient (*r* = 0.5) to account for within-person variability.

Two reviewers (Xing-Xian Li and Li Wang) independently assessed risk of bias using a modified Cochrane Risk of Bias Tool 1.0 (28, 29), including randomization sequence generation; allocation concealment; blinding of patients, healthcare providers, and outcome assessors; and incomplete outcome data (≥20% missing data was considered high risk of bias). Response options for each item were scored as “definitely or probably yes” (assigned a low risk of bias) or “definitely or probably no” (assigned a high risk of bias). Given that blinding of health care providers is generally not feasible in acupuncture trials, this domain was not considered when determining the overall risk of bias for each study (30). We classified a trial as having high risk of bias if any of the other five items was rated as high risk. Disagreements between reviewers were resolved by a third reviewer (Lei Lan).

### 2.4 Data synthesis

For studies with three arms, when one intervention group was shared across multiple comparisons, the shared group was evenly divided to avoid double-counting. For dichotomous outcomes, both the number of events and participants were divided, whereas for continuous outcomes, only the number of participants was divided, with the means and standard deviations left unchanged (31). When different instruments were used to measure the same outcome domain, the continuous measure was converted to the same scale on a domain-by-domain basis (32): (1) symptom relief was converted to a 195-point the Nepean Dyspepsia Symptom Index (NDSI) (higher score indicates worse symptom); (2) quality of life was converted to a 100-point Nepean Dyspepsia Life Quality Index (NDLQI) (higher score indicates better quality of life); (3) mental function was converted to 0–56 point Hamilton Anxiety Scale (HAMA) or 0–68 point Hamilton Depression Scale (HAMD) for anxiety or depression only, or 0–42 point Hospital Anxiety Depression Scale (HADS) for both together, with all indicating higher score for the worse mental function.

All data analyses were performed using a random-effect model with relative risk (RR) and the corresponding 95% confidence intervals (95% CI) for dichotomous outcomes and weighted mean differences (WMD) for continuous outcomes after converting to the same scales.

For binary outcomes with zero events, we applied a continuity correction by adding a constant of 0.5 to all cells in the 2 × 2 table for studies with zero events in one arm when pooling results using RR and 95% CI (31). We conducted sensitivity analyse using the Peto odds ratio (OR) method for outcomes (e.g., adverse events) with rare events, as recommended for sparse data. We also performed sensitivity analysis by applying alternative correlation coefficients (*r* = 0.25 and 0.75) compared to *r* = 0.5 in primary analysis for estimation of change scores to explore its impact on treatment effects.

The *Q* test and the *I*^2^ statistic were applied to assess statistical heterogeneity. Following Cochrane guidance, we evaluated *I*^2^ values as follows: 0–40% as “possibly not important,” 30–60% as “moderate heterogeneity,” 50–90% as “substantial heterogeneity,” and 75–100% as “considerable heterogeneity” (31). *A priori* subgroup analyses were conducted to explore potential sources of heterogeneity by assuming larger treatment effects were associated with: (1) manual acupuncture vs. electroacupuncture and (2) studies with high vs. low risk of bias. We conducted meta-regression to explore the association between treatment effect and length of follow up when there were at least 10 studies available (33)

Data were analyzed using Review Manager (RevMan), version 5.4.1; meta-regression and Egger’s test was performed using STATA software version 17. All comparisons were two-tailed using a threshold of *p* ≤ 0.05.

### 2.5 Certainty of evidence

We applied the GRADE methodology to assess the certainty of evidence for each outcome. Evidence can be downgraded from high to moderate, low, or very low certainty due to the risk of bias, consistency, directness, precision, and potential publication bias (34). Publication bias was assessed through a visual assessment of funnel plot asymmetry and Egger’s test for continuous outcomes and Harbord’s test for binary outcomes only when a meta-analysis included at least 10 studies. If we did not identify significant subgroup effects between studies at high vs. low risk of bias, we did not rate down the certainty of evidence for risk of bias. We considered pooled treatment effects as imprecise if the corresponding 95% CI crossed the null effect.

## 3 Results

### 3.1 Study characteristics

**Figure 1:**
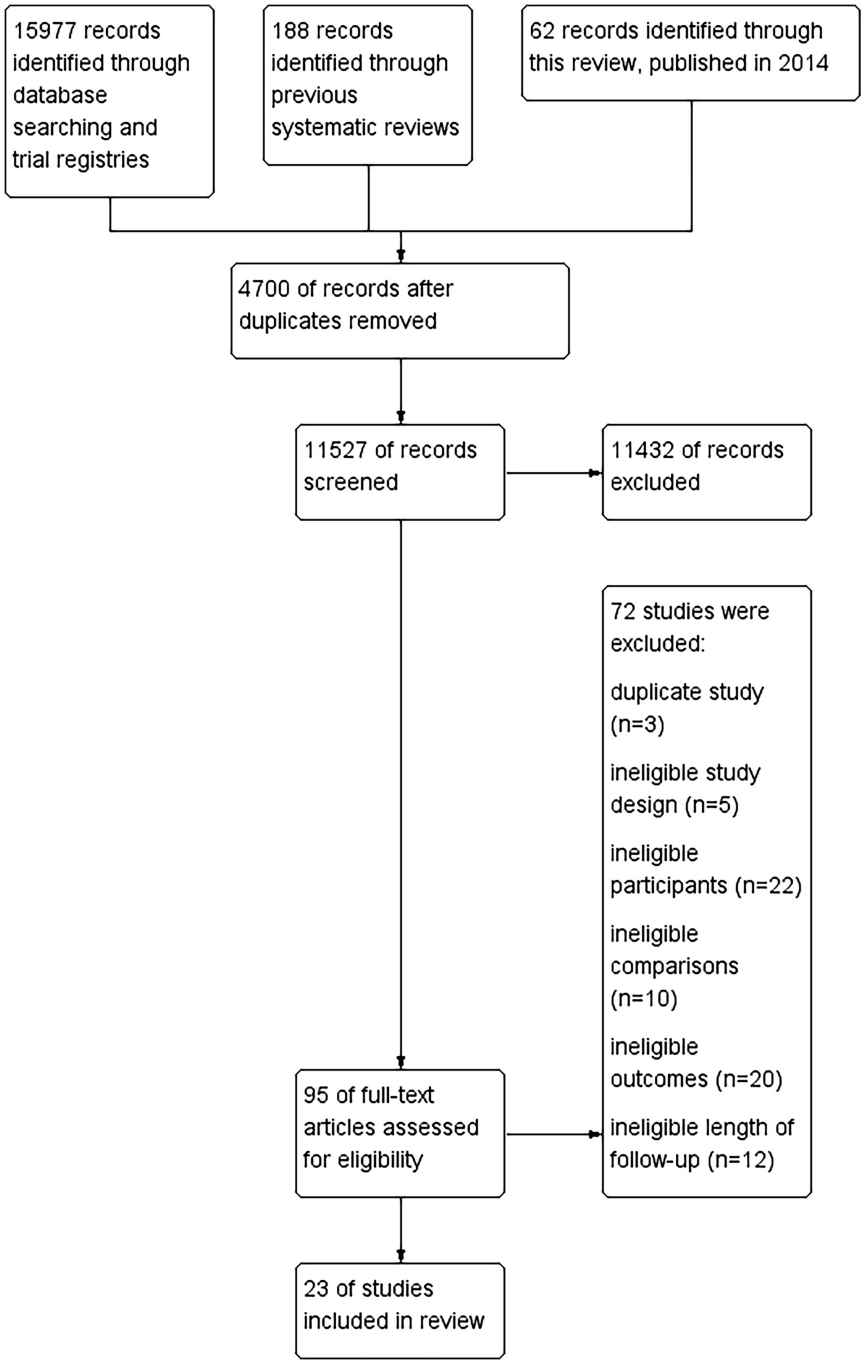
Flowchart of the search results and the selection details.

Of 15,977 citations were identified through a literature search, 23 studies (2,454 participants) were included (Figure 1). All studies were conducted in Asia, including 21 in China (35–55) and two in South Korea (56, 57). Twenty-two studies reported age with a median of the mean age of 42 years, and an interquartile range (IQR) of 37 to 45 years. Twenty-one studies provided sex distribution data with 1,517 (66%) female participants. The median of the mean duration of FD symptoms was 46 months (IQR 31 to 67 months) in 21 studies. Thirteen studies compared acupuncture with sham acupuncture (35, 38, 40–42, 46, 47, 49, 50, 52–55), four with no treatment or usual care (37, 45, 56, 57), and eight with pharmacotherapy, including mosapride (43, 48, 51), itopride (42, 55), rabeprazole and itopride (44), and domperidone (36, 39). Two three-arm trials compared electroacupuncture vs. sham electroacupuncture vs. itopride (42, 55). Thirteen were funded by government (35–38, 40–42, 47, 49, 50, 52, 56, 57), two by industry (45, 46), and eight without funding (39, 43, 44, 48, 51, 53–55) (Table 1).

**Table 1:** Baseline characteristics of included studies.

Study	Country	No. of participants	Duration of condition, months: mean (SD)	Age, years: mean (SD)	Female%	Intervention	Control	Length of follow-up (weeks)	Funding
Chang XR 2010 (35)	China	60	41 (20)	28 (6)	NR	Acupuncture	Sham acupuncture	4	Government
Chang Y 2023 (36)	China	60	32 (14)	43 (15)	58	Acupuncture	Domperidone	4	Government
Chung 2019 (37)	China	132	113 (115)	49 (12)	74	Electroacupuncture	No treatment	12	Government
Han XY 2024 (38)	China	70	29 (21)	42 (10)	61	Acupuncture	Sham acupuncture	4	Government
Kim MR 2019 (40)	China	51	51 (60)	42 (16)	73	Acupuncture	Sham acupuncture	4	Government
Ko SJ 2016 (56)	South Korean	76	145 (150)	49 (13)	70	Acupuncture	No treatment	4	Government
Lee B 2022 (57)	South Korean	20	NR	49 (11)	70	Acupuncture & usual care	Usual care	4	Government
Li DD 2014 (39)	China	70	78 (125)	37 (14)	67	Electroacupuncture	Domperidone	4	NR
Ma CY 2014 (41)	China	61	46 (8)	35 (5)	51	Electroacupuncture	Sham electroacupuncture	6	Government
Ma TT 2012 (42)	China	353	68 (72)	37 (13)	71	Electroacupuncture	Sham electroacupuncture; itopride	4	Government
Qiang LM 2018 (43)	China	64	53 (42)	45 (9)	59	Electroacupuncture	Mosapride	4	NR
Sheng JW 2013 (44)	China	100	NR	NR	NR	Electroacupuncture	Rabeprazole and itopride	4	NR
Tang KY 2023 (45)	China	84	16 (23)	44 (13)	69	Acupuncture	No treatment	4	Industrial
Tu JF 2020 (46)	China	42	45 (54)	45 (13)	67	Acupuncture	Sham acupuncture	4	Industrial
Yang JW 2020 (47)	China	278	60 (64)	41 (13)	67	Acupuncture	Sham acupuncture	4	Government
Yu F 2020 (48)	China	70	36 (18)	37 (8)	46	Acupuncture	Mosapride	4	NR
Zeng F 2012 (50)	China	64	40 (32)	24 (3)	61	Electroacupuncture	Sham electroacupuncture	4	Government
Zheng H 2018 (49)	China	200	31 (13)	39 (15)	68	Electroacupuncture	Sham electroacupuncture	16	Government
Zhou L 2019 (51)	China	60	21 (5)	36 (8)	47	Acupuncture	Mosapride	4	NR
Jin YL 2015 (52)	China	56	146 (134)	49 (11)	63	Acupuncture	Sham acupuncture	4	Government
Wang JJ 2015 (53)	China	68	7 (NR)	43 (NR)	74	Acupuncture	Sham acupuncture	4	NR
Yang ZQ 2011 (54)	China	61	65 (64)	28 (8)	56	Electroacupuncture	Sham electroacupuncture	4	NR
Yu SY 2010 (55)	China	354	67 (68)	38 (14)	67	Electroacupuncture	Sham electroacupuncture; itopride	4	NR

NR, not reported.

### 3.2 Risk of bias

Of 23 eligible trials, 20 (87%) adequately generated their randomization sequence, 13 (57%) adequately concealed allocation, 12 (52%) blinded participants, none blinded health care providers, 16 (70%) blinded outcome assessors, and all reported <20% missing outcome data (ranging from 0 to 18%) (Supplementary Table 2).

### 3.3 Acupuncture vs. sham acupuncture

#### 3.3.1 Symptom relief

**Figure 2:**
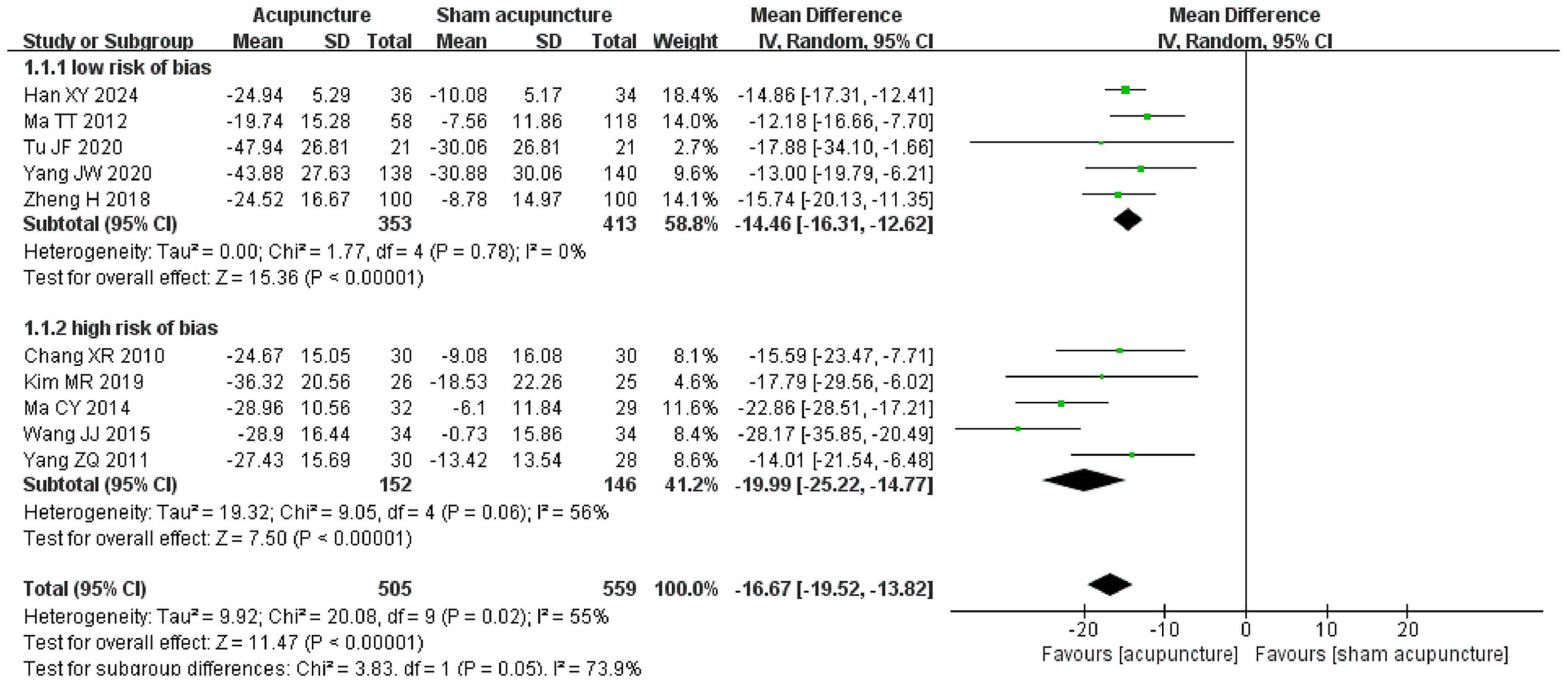
Subgroup analysis of symptom relief comparison between acupuncture and sham acupuncture, categorized by low and high risk of bias.

Significant subgroup effect was found between studies with high vs. low risk of bias (Figure 2, test of interaction *p* < 0.00001, moderate credibility, Supplementary Table 3) (35, 38, 40–42, 46, 47, 49, 53, 54). High certainty evidence from 5 RCTs (766 participants) with low risk of bias suggests that, compared to sham acupuncture, acupuncture reduces FD symptoms (WMD −14.46 points on the 195-point NDSI, 95% CI −16.31 to −12.62; Figure 2; Table 2) (38, 42, 46, 47, 49). We found no significant subgroup effect between studies with manual acupuncture vs. electroacupuncture (Supplementary Figure 1).

**Table 2:** Grade evidence profile of acupuncture versus sham acupuncture for people with functional dyspepsia.

No. of trials (no. of patients)	Follow-up, weeks	Risk of bias	Inconsistency	Indirectness	Imprecision	Publication bias	Treatment association (95% CI)	Overall quality of evidence
Symptom relief: 0 to 195 points NDSI for FD symptoms; lower is better								
5 (766)	4 to 16	Not serious ^a^	Not serious, *I*^2^ = 0%	Not serious	Not serious	NA	WMD −14.46 (−16.31, −12.62)	High
Quality of life: 0 to 100 points NDLQI for FD life quality; higher is better								
11 (1,240)	4 to 16	Not serious ^b^	Serious, *I*^2^ = 88%	Not serious	Not serious	Not serious	WMD 10.39 (7.06, 13.73)	Moderate
Anxiety and depression: 0 to 42 points HADS; lower is better								
2 (320)	4	Serious	Serious, *I*^2^ = 68%	Not serious	Serious ^c^	NA	WMD 0.00 (−3.50, 3.51)	Very low
Anxiety: 0 to 56 points HAMA; lower is better								
2 (126)	4	Serious	Serious, *I*^2^ = 86%	Not serious	Serious ^c^	NA	WMD −4.52 (−9.48, 0.45)	Very low
Depression: 0 to 68 points HAMD; lower is better								
1 (56)	4	Serious	NA	Not serious	Very serious ^d^	NA	WMD −7.95 (−12.85, −3.05)	Very low
Adverse effects								
5 (741)	4	Not serious ^b^	Not serious, *I*^2^ = 17%	Not serious	Serious ^c^	NA	RR 1.15 (0.63, 2.09)	Moderate

95% CI: 95% confidence interval; NDSI: Nepean dyspepsia symptom index; FD: functional dyspepsia; WMD: weighted mean difference; NDLQI: Nepean dyspepsia life quality index; NA, not available; HADS: hospital anxiety depression scale; HAMA: Hamilton anxiety scale; HAMD: Hamilton depression scale; RR: risk ratio.

a Moderate credible subgroup effect was found between studies with high vs. low risk of bias (test of interaction *p* < 0.00001); therefore, we only reported the pooed estimate among studies with low risk of bias.

b We did not rate down for risk of bias as we did not find significant subgroup effect between studies with high vs. low risk of bias.

c We rated down for imprecision because 95% CI crossed the null-effect line.

d Although the 95% CI did not cross the null-effect line, we rated down two levels for imprecision due to very small sample size.

#### 3.3.2 Quality of life

**Figure 3:**
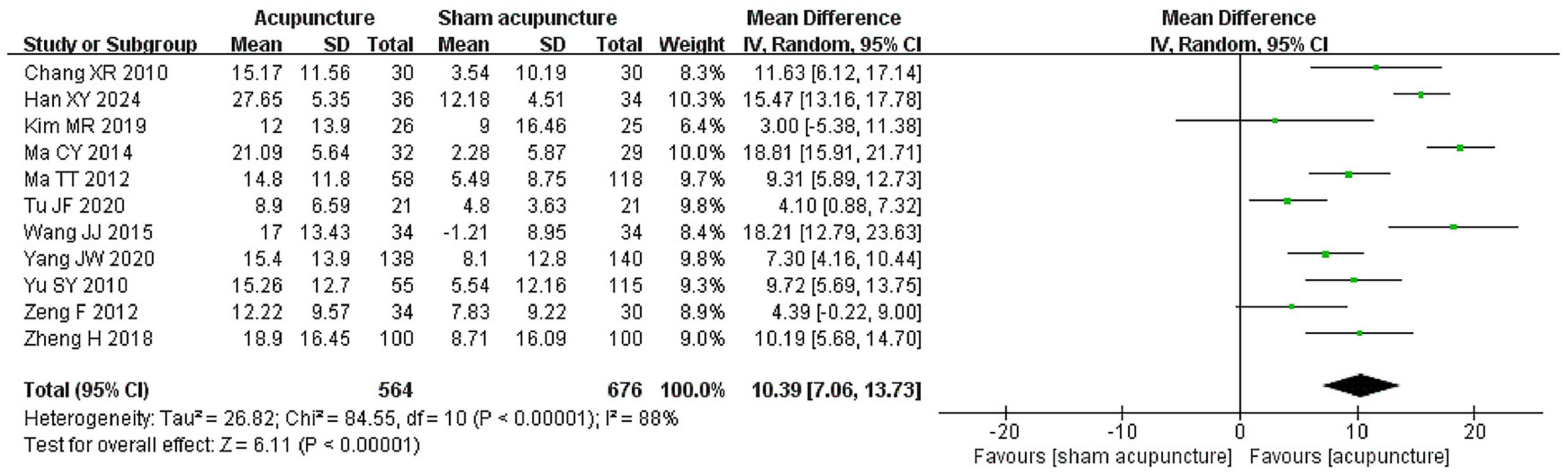
Quality of life among patients who received acupuncture compared to sham acupuncture.

Moderate certainty evidence from 11 RCTs (1,240 participants) suggests that, compared to sham acupuncture, acupuncture probably improves quality of life (WMD 10.39 points on the 100-point NDLQI, 95% CI 7.06 to 13.73; Figure 3; Table 2) (35, 38, 40–42, 46, 47, 49, 50, 53, 55). We found no significant subgroup effects between studies at high vs. low risk of bias (Supplementary Figure 2), or using manual acupuncture vs. electroacupuncture (Supplementary Figure 3). Meta-regression did not reveal significant association between quality of life and length of follow-up (*p* = 0.80, Supplementary Figure 4). No statistical evidence of publication bias was detected (Supplementary Figure 5 and Egger’s test *p* = 0.36).

#### 3.3.3 Mental functioning

Very low certainty evidence suggests that, compared to sham acupuncture, acupuncture might have little to no difference in improving anxiety and depression symptoms (2 RCTs, 320 participants, WMD 0.00 point on the 42-point HADS, 95% CI −3.50 to 3.51) (46, 47) or anxiety symptom only (2 RCTs, 126 participants, WMD −4.52 points on the 56-point HAMA, 95% CI −9.48 to 0.45) (38, 52), despite a small improvement in depression symptom from a single trial with very low certainty evidence (56 participants, WMD −7.95 points on the 68-point HAMD, 95% CI −12.85 to −3.05) (52) (Supplementary Figure 6; Table 2).

#### 3.3.4 Adverse effects

Moderate certainty evidence from 5 RCTs (741 participants) suggests probably little to no difference in adverse effects between acupuncture and sham acupuncture (RR 1.15, 95% CI 0.63 to 2.09; Supplementary Figure 7; Table 2) (40, 46, 47, 49, 55). No serious adverse effects were reported.

### 3.4 Acupuncture vs. no treatment or usual care

#### 3.4.1 Symptom relief

**Figure 4:**
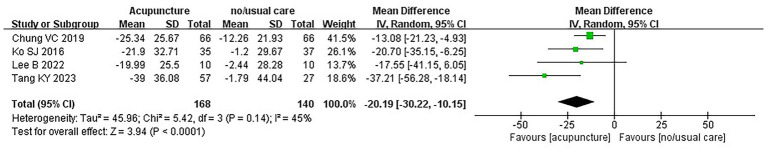
Symptom relief among patients who received acupuncture compared to no treatment or usual care.

Moderate certainty evidence from 4 RCTs (308 participants) suggests that, compared to no treatment or usual care, acupuncture probably improve FD symptoms (WMD −20.19 points on the 195-point NDSI, 95% CI −30.22 to −10.15; Figure 4; Supplementary Table 4) (37, 45, 56, 57).

#### 3.4.2 Quality of life

Very low certainty evidence from 3 RCTs (236 participants) suggests that, compared to no treatment or usual care, acupuncture may result in little to no difference in quality of life (WMD 15.02 points on the 100-point NDLQI, 95% CI −5.88 to 35.91; Supplementary Figure 8; Supplementary Table 4) (37, 45, 57).

#### 3.4.3 Mental functioning

Very low certainty evidence from one RCT (84 participants) suggests that, compared to no treatment or usual care, acupuncture may result in a small improvement in anxiety symptoms (WMD −12.69 points on the 56-point HAMA, 95% CI −17.06 to −8.32), but not in depression symptom (WMD −3.72 on the 68-point HAMD, 95% CI −9.71 to 2.27) (45) (Supplementary Table 4).

### 3.5 Acupuncture vs. pharmacotherapy

#### 3.5.1 Acupuncture vs. prokinetics drugs

Seven trials compared acupuncture with prokinetic drugs, including itopride (42, 55), mosapride (43, 48, 51), and domperidone (36, 39).

##### 3.5.1.1 Symptom relief

Low certainty evidence from 4 RCTs (381 participants) suggests that, compared to prokinetics drugs, acupuncture may improve FD symptoms (WMD −17.40 points on the 195-point NDSI, 95% CI −29.08 to −5.72; Supplementary Figure 9; Supplementary Table 5) (39, 42, 43, 48).

##### 3.5.1.2 Quality of life

**Figure 5:**
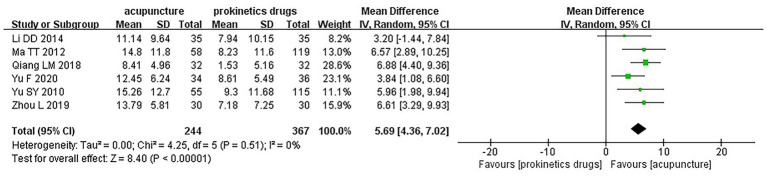
Quality of life among patients who received acupuncture compared to prokinetics drugs.

Moderate certainty evidence from 6 RCTs (611 participants) suggests that, compared to prokinetics drugs, acupuncture probably improves quality of life (WMD 5.69 points on the 100-point NDLQI, 95% CI 4.36 to 7.02; Figure 5; Supplementary Table 5) (39, 42, 43, 48, 51, 55). We found no subgroup effect between studies with manual acupuncture vs. electroacupuncture (Supplementary Figure 10).

##### 3.5.1.3 Mental functioning

Very low certainty evidence from one RCT (60 participants) suggests that, compared to domperidone, acupuncture may improve depression symptoms (WMD −5.36 points on the 68-point HAMD, 95% CI −8.58, −2.14), but may not for anxiety symptoms (WMD −2.54 points on the 56-point HAMA, 95% CI −5.23, 0.15) (36) (Supplementary Table 5).

##### 3.5.1.4 Adverse effects

Low certainty evidence from 2 RCTs (240 participants) suggests that, compared to prokinetics drugs, acupuncture may result in little to no difference in adverse effects (RR 1.31, 95% CI 0.29 to 6.00; Supplementary Figure 11; Supplementary Table 5) (48, 55). No serious adverse effects were reported.

#### 3.5.2 Acupuncture vs. Rabeprazole and Itopride

Very low certainty evidence from one RCT (100 participants) suggests that, compared to rabeprazole and itopride, acupuncture might result in improvement in both FD symptoms (WMD −11.09 points, 95% CI −16.52 to −5.66) and quality of life (WMD 7.98 points, 95% CI 3.93 to 12.03) with fewer adverse effects (RR 0.22, 95% CI 0.05 to 0.98) (44) (Supplementary Table 5).

### 3.6 Sensitivity analyses

Our sensitivity analyses using alternative correlation coefficients (*r* = 0.25 and 0.75) showed results consistent with the primary analysis using *r* = 0.5 (Supplementary Table 6). Our sensitivity analysis using Peto OR for adverse events outcomes showed consistent results (Supplementary Table 7).

## 4 Discussion

### 4.1 Overall findings

Our review with moderate- to high-certainty evidence found that, compared to sham acupuncture, acupuncture probably improves FD symptoms and quality of life without increasing the risk of adverse events, but its effects on anxiety and depression remain unclear. Compared to no treatment or usual care, acupuncture probably also improves FD symptoms and may improve anxiety symptoms. Compared to prokinetic drugs (mosapride, domperidone, and itopride), acupuncture probably improves quality of life and may reduce FD symptoms. Despite significant improvement in FD symptoms, quality of life and adverse event when compared acupuncture with the combination with itopride and rabeprazole, it was only supported by very low certainty evidence. We did not find significant subgroup effects between manual acupuncture and electroacupuncture in the treatment of FD across comparisons.

### 4.2 Relation to other studies

The most recent systematic review on acupuncture for improving symptoms of FD included 10 trials and found that acupuncture was superior to sham acupuncture and western medication regarding the improvements in FD symptoms and response rate (17). However, this review included 3 trials with very short follow-up (<2 weeks), and did not assess the certainty of evidence.

Our review incorporated 18 additional trials (35–42, 44–46, 48, 51, 53–57) offering a more comprehensive and updated synthesis. We confirmed the benefit of acupuncture over sham acupuncture in improving FD symptoms with high-certainty evidence. Moreover, we identify additional benefits with moderate certainty evidence in quality of life when compared to sham acupuncture or prokinetic drugs, and in FD symptom relief when compared to no treatment or usual care.

Despite these encouraging results, the comparative effectiveness of acupuncture on anxiety, depression, and adverse events remains uncertain due to limited and inconsistent data. This highlights the need for future trials with robust mental health assessments and longer follow-up periods to clarify acupuncture’s role in addressing the psychological dimensions of FD.

Our review also found no significant subgroup differences between manual acupuncture and electroacupuncture across comparisons, suggesting that both modalities may be equally effective in clinical practice. This finding offers flexibility for practitioners and patients when selecting acupuncture techniques based on preference, availability, or cost considerations.

### 4.3 Strengths and limitations

We conducted a comprehensive search for eligible RCTs in any language. We used *a priori* subgroup analyses to explore the source of heterogeneity and assessed the credibility of potential subgroup effects. We also applied the GRADE approach to assess the certainty of the evidence for each outcome, providing a transparent and standardized assessment of evidence quality and strengthening the reliability and clinical interpretability of our conclusions.

However, there are several limitations in this review. First, many included studies are at high risk of bias due to inadequate blinding of patients, health care providers, or outcome assessor, as well as allocation concealment. Second, all included trials were conducted in Asia, which may limit the generalizability of the findings to other populations. Third, few trials compared acupuncture with active treatments, e.g., pharmacotherapy, restricting conclusions about its relative effectiveness.

### 4.4 Implications

We identified 23 RCTs that assessed the efficacy and safety of acupuncture in the treatment of FD. Moderate to high certainty evidence suggests that acupuncture likely improves FD symptoms and quality of life compared to sham acupuncture, probably improves FD symptoms compared to no treatment or usual care, and probably improves quality of life compared to prokinetics. However, there is a lack of evidence on the benefits in psychological dimension among FD patients.

To strengthen the clinical applicability of our findings, we summarized the implementation characteristics of acupuncture interventions across the included trials in Supplementary Table 8. Most interventions involved manual acupuncture or electroacupuncture targeting core points such as ST36 (Zusanli), CV12 (Zhongwan), and PC6 (Neiguan). Treatment protocols typically consisted of 3–5 sessions per week over approximately 4 weeks, with individual sessions lasting 20–30 min. These patterns suggest that a moderate-frequency regimen emphasizing the stomach meridian and related points may be clinically relevant for functional dyspepsia management. The evidence primarily pertains to patients diagnosed according to Rome III or IV criteria, particularly those with the postprandial distress syndrome (PDS) subtype, aged 30–60 years, and without severe comorbidities. However, evidence remains limited for older adult patients, the epigastric pain syndrome (EPS) subtype, and those with psychiatric comorbidities, highlighting important gaps for future research.

Future trials should also prioritize methodological rigor, including adequate randomization, allocation concealment, and blinding to investigate the comparative effectiveness between acupuncture and active treatments for FD.

Our findings support the integration of acupuncture as a viable therapeutic option for FD, particularly for symptom relief and quality of life enhancement. Adverse events associated with acupuncture for FD appear to be rare. Given its favorable effectiveness and safety profile, acupuncture could be considered as a complementary or alternative approach in settings where conventional pharmacologic treatments are ineffective, poorly tolerated, or contraindicated. While these findings are promising, the comparative effectiveness of acupuncture for anxiety, depression, and adverse events remains inconclusive due to mostly very low to low certainty evidence. Consequently, clinicians should cautiously interpret the implications for psychological outcomes and adverse event profiles.

## 5 Conclusion

Compared to sham acupuncture, acupuncture probably improves FD symptoms and quality of life without significant increase in adverse events. It probably also improve FD symptoms when compared to no treatment or usual care. In comparisons with prokinetic drugs such as mosapride, domperidone, and itopride, acupuncture probably enhances quality of life and may reduce FD symptoms. However, its effects on anxiety and depression, as well as its comparative effectiveness against other pharmacological or non- pharmacological treatments remain uncertain.

Additionally, manual acupuncture and electroacupuncture appear to have comparable effects in the treatment of FD, suggesting that either modality may be selected based on patient preference and resource availability. Nonetheless, head-to-head comparisons between these two acupuncture techniques, as well as acupuncture and active treatments are needed to better understand its comparative effects in managing FD.

## Data Availability

The original contributions presented in the study are included in the article/Supplementary material, further inquiries can be directed to the corresponding authors.

## Footnotes

1
https://doi.org/10.1002/14651858.CD008487
