# Supplementary material for: Efficacy and safety of acupuncture for functional dyspepsia: an updated meta-analysis of randomized controlled trials
Source: Front Med (Lausanne). 2026 Feb 9;13:1718632. doi: 10.3389/fmed.2026.1718632 (PMC12926150; doi:10.3389/fmed.2026.1718632)
Supplement: Supplementary file 1 [file Table_1.docx]

**Supplement Table 1. Literature Search Strategies in Databases**

**Supplement Table 1.1 Pubmed search <inception to March 13, 2025>**

| **Search Strategy and Search Results** |
| --- |
| **Inception to February 3, 2023** |
| #1 Dyspepsia [MeSH Terms] (9167)  #2 indigestion OR postprandial distress syndrome OR epigastric pain syndrome OR functional dyspepsia (5053)  #3 functional gastrointestinal disorders OR functional gastrointestinal dis* (3222)  #4 burning or inappetence or epigastric or discomfort or epigastric or pain or abdominal or distension or early or satiety or belching (2785563)  #5 #1 OR #2 OR #3 OR #4 (2795535)  #6 Acupuncture [MeSH Terms] (29287)  #7 Acupuncture Therapy [MeSH Terms] (28520)  #8 acupuncture treatment* OR electroacupuncture OR meridians OR acupunctur* (31567)  #9 Meridians OR Auriculotherapy OR Acupoint* OR Jing Luo OR Luo, Jing OR Jingluo OR acupunctur* OR acu-point* (30978)  #10 #6 OR #7 OR #8 OR #9 (38682)  #11 #5 AND #10 (10597)  #12 "inception-current"[Date - Publication] (9164) |
| **February 1, 2023 to December 31, 2023** |
| #1 Dyspepsia [MeSH Terms] (9325)  #2 indigestion OR postprandial distress syndrome OR epigastric pain syndrome OR functional dyspepsia (5315)  #3 functional gastrointestinal disorders OR functional gastrointestinal dis* (3405)  #4 burning or inappetence or epigastric or discomfort or epigastric or pain or abdominal or distension or early or satiety or belching (2925446)  #5 #1 OR #2 OR #3 OR #4 (2935673)  #6 Acupuncture [MeSH Terms] (30438)  #7 Acupuncture Therapy [MeSH Terms] (29667)  #8 acupuncture treatment* OR electroacupuncture OR meridians OR acupunctur* (33372)  #9 Meridians OR Auriculotherapy OR Acupoint* OR Jing Luo OR Luo, Jing OR Jingluo OR acupunctur* OR acu-point* (32734)  #10 #6 OR #7 OR #8 OR #9 (40748)  #11 #5 AND #10 (11264)  #12 "2023/02/01"[Date - Publication] : "2023/12/31"[Date - Publication]] (771) |
| **January 1, 2024 to March 13, 2025** |
| #1 Dyspepsia [MeSH Terms] (9522)  #2 indigestion OR postprandial distress syndrome OR epigastric pain syndrome OR functional dyspepsia (5719)  #3 functional gastrointestinal disorders OR functional gastrointestinal dis* (3680)  #4 burning or inappetence or epigastric or discomfort or epigastric or pain or abdominal or distension or early or satiety or belching (3151166)  #5 #1 OR #2 OR #3 OR #4 (3161813)  #6 Acupuncture [MeSH Terms] (32239)  #7 Acupuncture Therapy [MeSH Terms] (31463)  #8 acupuncture treatment* OR electroacupuncture OR meridians OR acupunctur* (36227)  #9 Meridians OR Auriculotherapy OR Acupoint* OR Jing Luo OR Luo, Jing OR Jingluo OR acupunctur* OR acu-point* (35511)  #10 #6 OR #7 OR #8 OR #9 (44010)  #11 #5 AND #10 (12391)  #12 "2024/01/01"[Date - Publication] : "2025/03/13"[Date - Publication]] (1117) |

**Supplement Table 1.2 Embase search <inception to March 13, 2025>**

| **Search Strategy and Search Results** |
| --- |
| **Inception to February 3, 2023** |
| #1 dyspepsia (42594)  #2 indigestion (4014)  #3 #1 OR #2 (46113)  #4 'acupuncture point' OR 'acupuncture needle' OR 'acupuncture' (65929)  #5 meridian OR 'Jing luo' OR Jingluo (14475)  #6 electrostimulation (93905)  #7 transcutaneous electrical nerve stimulation (10262)  #8 #4 OR #5 OR #6 OR #7 (174,993)  #9 #3 AND #8 (721)  #10 limit #9 to (human AND yr="inception -Current") (636) |
| **January 1, 2023 to December 31, 2023** |
| #1 dyspepsia (44287)  #2 indigestion (4250)  #3 #1 OR #2 (48008)  #4 'acupuncture point' OR 'acupuncture needle' OR 'acupuncture' (69833)  #5 meridian OR 'Jing luo' OR Jingluo (15203)  #6 electrostimulation (96621)  #7 transcutaneous electrical nerve stimulation (10907)  #8 #4 OR #5 OR #6 OR #7 (198,430)  #9 #3 AND #8 (764)  #10 limit #9 to (human AND yr="2023 -2023") (44) |
| **January 1, 2024 to March 13, 2025** |
| #1 dyspepsia (46650)  #2 indigestion (4585)  #3 #1 OR #2 (50673)  #4 'acupuncture point' OR 'acupuncture needle' OR 'acupuncture' (76714)  #5 meridian OR 'Jing luo' OR Jingluo (36056)  #6 electrostimulation (100449)  #7 transcutaneous electrical nerve stimulation (12582)  #8 #4 OR #5 OR #6 OR #7 (214,249)  #9 #3 AND #8 (869)  #10 limit #9 to (human AND yr="2024 -2025") (88) |
|  |

**Supplement Table 1.3 Cochrane Library search <inception to March 13, 2025>**

| **Search Strategy and Search Results** |
| --- |
| **Inception to February 3, 2023** |
| #1 Dyspepsia* in Cochrane Reviews, Trials (5740)  #2 functional dyspepsia (2250)  #3 Indigestion (686)  #4 postprandial distress syndrome (176)  #5 Functional Gastrointestinal Dis* (7172)  #6 FGID* (132)  #7 FD*(16944)  #8 burning or inappetence (10667)  #9 epigastric (2007)  #10 distension (3785)  #11 belching (395)  #12 {OR #1-#11} (43904)  #13 Acupuncture Therapy (9764)  #14 Electroacupuncture (3135)  #15 Acupuncture Points (6612)  #16 Meridians (1285)  #17 Acupoint (4890)  #18 Jing Luo (4)  #19 Jingluo (2)  #20 acupunctur* (17684)  #21 Transcutaneous Electric Nerve Stimulation (3471)  #22 TENS (35054)  #23 {OR #13-#22} (56430)  #24 {AND #12, #23} Limits date from inception to current(1705) |
| **February 1, 2023 to December 31, 2023** |
| #1 Dyspepsia* in Cochrane Reviews, Trials (6289)  #2 functional dyspepsia (2681)  #3 Indigestion (864)  #4 postprandial distress syndrome (234)  #5 Functional Gastrointestinal Dis* (10489)  #6 FGID* (152)  #7 FD*(22497)  #8 burning or inappetence (14895)  #9 epigastric (2337)  #10 distension (4376)  #11 belching (718)  #12 {OR #1-#11} (56929)  #13 Acupuncture Therapy (11931)  #14 Electroacupuncture (3621)  #15 Acupuncture Points (8229)  #16 Meridians (1835)  #17 Acupoint (5762)  #18 Jing Luo (125)  #19 Jingluo (4)  #20 acupunctur* (21495)  #21 Transcutaneous Electric Nerve Stimulation (4100)  #22 TENS (42102)  #23 {OR #13-#22} (67184)  #24 {AND #12, #23} (3732)  #25 Limits date between Jan 2023 and Dec 2023 {#24} (320) |
| **January 1, 2024 to March 13, 2025** |
| #1 Dyspepsia* in Cochrane Reviews, Trials (6397)  #2 functional dyspepsia (2812)  #3 Indigestion (937)  #4 postprandial distress syndrome (260)  #5 Functional Gastrointestinal Dis* (11283)  #6 FGID* (167)  #7 FD*(24415)  #8 burning or inappetence (16009)  #9 epigastric (2486)  #10 distension (4575)  #11 belching (770)  #12 {OR #1-#11} (60973)  #13 Acupuncture Therapy (13019)  #14 Electroacupuncture (3993)  #15 Acupuncture Points (9050)  #16 Meridians (1976)  #17 Acupoint (6452)  #18 Jing Luo (128)  #19 Jingluo (4)  #20 acupunctur* (23287)  #21 Transcutaneous Electric Nerve Stimulation (4598)  #22 TENS (44165)  #23 {OR #13-#22} (71642)  #24 {AND #12, #23} (3986)  #25 Limits date between Jan 2024 and Mar 2025 {#24} (357) |
|  |

**Supplement Table 1.4 CNKI/Wan Fang/VIP search <inception to March 13, 2025>**

| **Search Strategy and Search Results** |
| --- |
| #1 Dyspepsia OR functional dyspepsia  #2 Acupuncture OR electro-acupuncture  #3 Clinical randomized controlled trial OR randomized control OR randomized OR RCT  #4 #1 AND #2 AND #3  #5 "until 2025-03-13"[Date - Publication]  #6 #4 AND #5  #1 消化不良 + 功能性消化不良 + 肠易激综合征 + 便秘 + 功能性便秘  #2 针灸 + 针刺疗法 + 电针 + 针刺治疗 + 针刺  #3 临床随机对照试验 + 随机对照 + 随机对照试验 + RCT  #4 #1 AND #2 AND #3  #5 时间范围：建库——2025年3月13日  #6 #4 AND #5 |

**Supplement Table 1.5 Clinicaltrials.gov search <inception to March 13, 2025>**

| **Search Strategy and Search Results** |
| --- |

Condition or disease: functional dyspepsia

Other terms: dyspepsia

Intervention: acupuncture OR electroacupuncture

Study type: interventional studies (clinical trials)

Study start From 01/01/1900 To 03/13/2025

(8)

**Supplement Table 1.6 WHO ICTRP search <inception to March 13, 2025>**

| **Search Strategy and Search Results** |
| --- |

In the Condition: functional dyspepsia or dyspepsia

In the Intervention: acupuncture OR electroacupuncture

| Date of registration is between 01/01/1900 and 13/03/2025 |
| --- |

(15)
