# Supplementary material for: Efficacy and safety of acupuncture for functional dyspepsia: an updated meta-analysis of randomized controlled trials
Source: Front Med (Lausanne). 2026 Feb 9;13:1718632. doi: 10.3389/fmed.2026.1718632 (PMC12926150; doi:10.3389/fmed.2026.1718632)
Supplement: Supplementary file 2 [file Table_2.docx]

**Supplement Table 2. Risk of bias of included studies**

| **Study** | **Adequate randomization sequence generation** | **Adequate allocation concealment** | **Blinding of patients** | **Blinding of health care providers** | **Blinding of outcome assessors** | **Loss to follow-up (%)** |
| --- | --- | --- | --- | --- | --- | --- |
| Chang XR 2010 | High | High | Low | High | Low | 0 |
| Chang Y 2023 | Low | High | High | High | High | 0 |
| Chung 2019 | Low | Low | High | High | Low | 0 |
| Kim MR 2019 | Low | High | Low | High | Low | 17.74 |
| Ko SJ 2016 | Low | Low | High | High | High | 5.26 |
| Lee B 2022 | Low | Low | High | High | Low | 16.67 |
| Li DD 2014 | Low | High | High | High | High | 0 |
| Ma CY 2014 | Low | High | Low | High | Low | 14.75 |
| Ma TT 2012 | Low | Low | Low | High | Low | 1.12 |
| Qiang LM 2018 | Low | High | High | High | High | 0 |
| Sheng JW 2013 | High | High | High | High | High | 0 |
| Tang KY 2023 | Low | High | High | High | High | 6.67 |
| Tu JF 2020 | Low | Low | Low | High | Low | 0 |
| Yang JW 2020 | Low | Low | Low | High | Low | 0 |
| Yu F 2020 | Low | Low | High | High | Low | 2.78 |
| Zeng F 2012 | Low | Low | Low | High | Low | 11.11 |
| Zheng H 2018 | Low | Low | Low | High | Low | 2 |
| Zhou L 2019 | Low | Low | High | High | High | 0 |
| Han XY 2024 | Low | Low | Low | High | Low | 10.26 |
| Wang JJ 2015 | Low | High | Low | High | Low | 0 |
| Yang ZQ 2011 | High | High | Low | High | Low | 4.92 |
| Jin YL 2015 | Low | Low | Low | High | Low | 6.67 |
| Yu SY 2010 | Low | Low | High | High | Low | 4 |
