# Supplementary material for: Efficacy and safety of acupuncture for functional dyspepsia: an updated meta-analysis of randomized controlled trials
Source: Front Med (Lausanne). 2026 Feb 9;13:1718632. doi: 10.3389/fmed.2026.1718632 (PMC12926150; doi:10.3389/fmed.2026.1718632)
Supplement: Supplementary file 3 [file Table_3.docx]

Supplementary Table 3 ICEMAN criteria for assessing the credibility of subgroup effect of low vs. high risk of bias for FD symptom between acupuncture vs. sham acupuncture

| **Criteria** | **Subgroup effect** |
| --- | --- |
| 1: Is the analysis of effect modification based on comparison within rather than between trials? | Between-study |
| 2: For within-trial comparisons, is the effect modification similar from trial to trial? | Not applicable |
| 3: For between-trial comparisons, is the number of trials large? | **Probably large (5 vs. 5 studies for low vs. high risk of bias)** |
| 4: Was the direction of effect modification correctly hypothesized a priori? | **Definitely yes** |
| 5: Does a test for interaction suggest that chance is an unlikely explanation of the apparent effect modification? | **Chance an unlikely explanation (p<0.00001)** |
| 6: Did the authors test only a small number of effect modifiers or consider the number in their statistical analysis? | **Definitely yes** |
| 7: Did the authors use a random effects model? | **Definitely yes** |
| 8: If the effect modifier is a continuous variable, were arbitrary cut points avoided? | NA |
| 9 Optional: Are there any additional considerations that may increase or decrease credibility? | NA |
| Overall credibility | **Moderate** |

ICEMAN: Instrument for assessing the Credibility of Effect Modification Analyses; FD: functional dyspepsia; NA: not applicable
