# Supplementary material for: Efficacy and safety of acupuncture for functional dyspepsia: an updated meta-analysis of randomized controlled trials
Source: Front Med (Lausanne). 2026 Feb 9;13:1718632. doi: 10.3389/fmed.2026.1718632 (PMC12926150; doi:10.3389/fmed.2026.1718632)
Supplement: Supplementary file 4 [file Table_4.docx]

Supplement Table 4. Grade evidence profile of acupuncture versus no treatment or usual care for people with functional dyspepsia

| **No. of trials**  **(No. of patients)** | **Follow-up, weeks** | **Risk of bias** | **Inconsistency** | **Indirectness** | **Imprecision** | | **Publication bias** | | | **Treatment association (95% CI)** | | | **Overall quality of evidence** | |  |  |  |  |  |  |  |  |
| --- | --- | --- | --- | --- | --- | --- | --- | --- | --- | --- | --- | --- | --- | --- | --- | --- | --- | --- | --- | --- | --- | --- |
| **Symptom relief: 0 to 195 points NDSI for FD symptoms; lower is better** | | | | | | | | | | | | | | |  |  |  |  |  |  |  |  |
| 4 (308) | 4 to 12 | Serious ^a^ | Not serious, I^2^=45% | Not serious | | Not serious | | NA | | | WMD -20.19 (-30.22, -10.15) | | | Moderate | |  |  |  |  |  |  |  |
| **Quality of life: 0 to 100 points NDLQI for FD life quality; higher is better** | | | | | | | | | | | | | | |  |  |  |  |  |  |  |  |
| 3 (236) | 4 to 12 | Serious ^a^ | Serious, I^2^=95% | Not serious | | Serious^b^ | | | NA | | | WMD 15.02 (-5.88, 35.91) | Very Low | | | |  |  |  |  |  |  |
| **Anxiety: 0 to 56 points HAMA; lower is better** | | | | | | | | | | | | | | |  | | |  |  |  |  |  |
| 1 (84) | 4 | Serious ^a^ | NA | Not serious | | Very serious ^c^ | | NA | | | WMD -12.69 (-17.06, -8.32) | | | Very Low | |  |  |  |  |  |  |  |
| **Depression: 0 to 68 points HAMD; lower is better** | | | | | | | | | | | | | | | |  |  |  |  |  |  |  |
| 1 (84) | 4 | Serious ^a^ | NA | Not serious | | Very serious ^b,c^ | | NA | | | WMD -3.72 (-9.71, 2.27) | | | Very Low | |  |  |  |  |  |  |  |

Abbreviations: 95% confidence interval; NDSI: Nepean Dyspepsia Symptom Index; FD: functional dyspepsia; WMD: weighted mean difference; NDLQI: Nepean Dyspepsia Life Quality Index; NA, not available; HADS: Hospital Anxiety Depression Scale; HAMA: Hamilton Anxiety Scale; HAMD: Hamilton Depression Scale.

a. high risk of bias in blinding;

b. We rated down for imprecision because 95% CI crossed the null-effect line;

c. We rated down two levels for imprecision due to very small sample size.
