# Supplementary material for: Efficacy and safety of acupuncture for functional dyspepsia: an updated meta-analysis of randomized controlled trials
Source: Front Med (Lausanne). 2026 Feb 9;13:1718632. doi: 10.3389/fmed.2026.1718632 (PMC12926150; doi:10.3389/fmed.2026.1718632)
Supplement: Supplementary file 5 [file Table_5.docx]

Supplement Table 5. Grade evidence profile of acupuncture versus medications for people with functional dyspepsia

| **Comparison** | **No. of trials**  **(No. of patients)** | **Follow-up, week** | **Risk of bias** | **Inconsistency** | **Indirectness** | **Imprecision** | **Publication bias** | **Treatment association (95% CI)** | **Overall quality of evidence** |  |
| --- | --- | --- | --- | --- | --- | --- | --- | --- | --- | --- |
| Acupuncture vs. prokinetic Drugs | **Symptom relief: 0 to 195 points NDSI for FD symptoms; lower is better** | | | | | | | | |  |
|  | 4 (381) | 4 | Serious ^a^ | Serious, I^2^=95% | Not serious | Not serious | NA | WMD -17.40 (-29.08, -5.72) | Low |  |
|  | **Quality of life: 0 to 100 points NDLQI for FD life quality; higher is better** | | | | | | | | |  |
|  | 6 (611) | 4 | Serious ^a^ | Not serious, I^2^=0% | Not serious | Not serious | NA | WMD 5.69 (4.36, 7.02) | Moderate |  |
|  | **Anxiety: 0 to 56 points HAMA; lower is better** | | | | | | | | |  |
|  | 1 (60) | 4 | Serious ^a^ | NA | Not serious | Very serious ^b^ | NA | WMD -2.54 (-5.23, 0.15) | Very Low |  |
|  | **Depression: 0 to 68 points HAMD; lower is better** | | | | | | | | |  |
|  | 1 (60) | 4 | Serious ^a^ | NA | Not serious | Very serious ^c^ | NA | WMD -5.36 (-8.58, -2.14) | Very Low |  |
|  | **Adverse effects** | | | | | | | | |  |
|  | 2 (240) | 4 | Serious ^a^ | Not serious, I^2^=0% | Not serious | Serious ^d^ | NA | RR 1.31 (0.29, 6.00) | Low |  |
| Acupuncture vs. Rabeprazole and Itopride | **Symptom relief: 0 to 195 points NDSI for FD symptoms; lower is better** | | | | | | | | |  |
|  | 1 (100) | 4 | Serious ^e^ | NA | Not serious | Very serious ^c^ | NA | WMD -11.09 (-16.52, -5.66) | Very Low |  |
|  |  |  |  |  |  |  |  |  |  |  |
|  | **Quality of life: 0 to 100 points NDLQI for FD life quality; higher is better** | | | | | | | | |  |
|  | 1 (100) | 4 | Serious ^e^ | NA | Not serious | Very serious ^c^ | NA | WMD 7.98 (3.93, 12.03) | Very Low |  |
|  |  |  |  |  |  |  |  |  |  |  |
|  | **Adverse effects** | | | | | | | | |  |
|  | 1 (100) | 4 | Serious ^e^ | NA | Not serious | Very serious ^c^ | NA | RR 0.22 (0.05, 0.98) | Very Low |  |

Abbreviations: 95% CI: 95% confidence interval; NDSI: Nepean Dyspepsia Symptom Index; FD: functional dyspepsia; WMD: weighted mean difference; NDLQI: Nepean Dyspepsia Life Quality Index; NA, not available; RR: Risk Ratio; HADS: Hospital Anxiety Depression Scale; HAMA: Hamilton Anxiety Scale; HAMD: Hamilton Depression Scale.

1. High risk of bias in blinding;
2. We rated down two levels for imprecision because very small sample size and the 95% CI crossed the null-effect line;
3. Although the 95% CI didn’t cross the null-effect line, we rated down two levels for imprecision due to very small sample size.
4. We rated down one level for imprecision because the 95% CI crossed the null-effect line.
5. High risk of bias in blinding and randomization
