# Supplementary material for: Efficacy and safety of acupuncture for functional dyspepsia: an updated meta-analysis of randomized controlled trials
Source: Front Med (Lausanne). 2026 Feb 9;13:1718632. doi: 10.3389/fmed.2026.1718632 (PMC12926150; doi:10.3389/fmed.2026.1718632)
Supplement: Supplementary file 6 [file Table_6.docx]

**Supplement Table 6. Sensitivity analyses using correlation coefficients of r=0.25 and 0.75 vs. primary analysis using r=0.5**

| **r** | **(WMD, 95%CI)** | **P** | **I²** |
| --- | --- | --- | --- |
| **Symptom relief when acupuncture vs. sham acupuncture** | | | |
| 0.25 | -14.29,[-16.37,-12.21] | ＜0.00001 | 0% |
| 0.5 | -14.46,[-16.31,-12.62] | ＜0.00001 | 0% |
| 0.75 | -14.68,[-16.19,-13.18] | ＜0.00001 | 0% |
| **Quality of life when acupuncture vs. sham acupuncture** | | | |
| 0.25 | 10.41,[7.08,13.74] | ＜0.00001 | 85% |
| 0.5 | 10.39,[7.06,13.73] | ＜0.00001 | 88% |
| 0.75 | 10.39,[7.06,13.73] | ＜0.00001 | 88% |
| **Symptom relief when acupuncture vs. no treatment or usual care** | | | |
| 0.25 | -18.71,[-28.26,-9.15] | 0.0001 | 26% |
| 0.5 | -20.19,[-30.22,-10.15] | ＜0.0001 | 45% |
| 0.75 | -21.37,[-31.32,-11.42] | ＜0.0001 | 65% |
| **Quality of life when acupuncture vs. no treatment or usual care** | | | |
| 0.25 | 15.22, [-6.55,36.99] | 0.17 | 95% |
| 0.5 | 15.02, [-5.88,35.91] | 0.16 | 95% |
| 0.75 | 14.77, [-4.38,33.91] | 0.13 | 95% |
| **Symptom relief when acupuncture vs. Prokinetics Drugs** | | | |
| 0.25 | -16.77, [-27.89,-5.66] | 0.003 | 93% |
| 0.5 | -17.40, [-29.08,-5.72] | 0.003 | 95% |
| 0.75 | -18.02, [-30.91,-5.14] | 0.006 | 97% |
| **Quality of life when acupuncture vs. Prokinetics Drugs** | | | |
| 0.25 | 5.72,[4.16,7.28] | ＜0.00001 | 0% |
| 0.5 | 5.69,[4.36,7.02] | ＜0.00001 | 0% |
| 0.75 | 5.63,[4.35,6.91] | ＜0.00001 | 34% |

Abbreviations: 95% CI: 95% confidence interval; WMD: weighted mean difference
