# Supplementary material for: Efficacy and safety of acupuncture for functional dyspepsia: an updated meta-analysis of randomized controlled trials
Source: Front Med (Lausanne). 2026 Feb 9;13:1718632. doi: 10.3389/fmed.2026.1718632 (PMC12926150; doi:10.3389/fmed.2026.1718632)
Supplement: Supplementary file 7 [file Table_7.docx]

**Supplement Table 7. Sensitivity analysis for adverse effects**

| **Outcome** | **Comparison** | **RR (95% CI)** | **Peto OR (95% CI)** |
| --- | --- | --- | --- |
| Adverse effects | Acupuncture Vs. Sham Acupuncture | 1.15 (0.63, 2.09) | 1.20 (0.69, 2.07) |
| Adverse effects | Acupuncture Vs. Prokinetics Drugs | 1.31 (0.29, 6.00) | 1.19 (0.23, 6.28) |

Abbreviations: RR: risk ratio; 95% CI: 95% confidence interval; Peto OR: Peto Odds Ratio
