# Supplementary material for: Efficacy and safety of acupuncture for functional dyspepsia: an updated meta-analysis of randomized controlled trials
Source: Front Med (Lausanne). 2026 Feb 9;13:1718632. doi: 10.3389/fmed.2026.1718632 (PMC12926150; doi:10.3389/fmed.2026.1718632)
Supplement: Supplementary file 8 [file Table_8.docx]

| **Supplement Table 8. Summary of Acupuncture Interventions** | | | | | |
| --- | --- | --- | --- | --- | --- |
| **Study** | **Diagnostic Criteria** | **Intervention Type** | **Primary Acupoints** | **Treatment Duration per Session** | **Treatment Frequency & Duration** |
| Chang XR 2010(35) | Rome Ⅲ | manual acupuncture | ST40, ST36, ST34, ST42 | 30 min | 5 times/week × 4weeks |
| Chang Y 2023(36) | Rome Ⅳ | manual acupuncture | CV12, ST36, PC6, LR3, GV20, GV24, EX-HN3, HT7 | 30 min | 6 times/week × 4weeks |
| Chung 2019(37) | Rome Ⅳ,  PDS | electroacupuncture,  2 Hz continuous wave; intensity 0.5–1.5 mA | ST34, ST36, ST40, ST42, CV12, PC6 | 30 min | 2 times/week × 10weeks |
| Han XY 2024(38) | Rome Ⅳ | manual acupuncture | PC6, CV12, ST36, ST25 | 30 min | 6 times/week × 4weeks |
| Kim MR 2019(40) | Rome Ⅳ,  PDS | manual acupuncture | GV20, CV12, CV6, ST25, PC6, ST36, SP4, CV17 | 20 min | 3 times/week × 4weeks |
| Ko SJ 2016(56) | Rome Ⅲ | manual acupuncture | LI4, ST36, LR3, SP4, CV12 | 15 min | 2 times/week × 4weeks |
| Lee B 2022(57) | Rome Ⅲ | manual acupuncture | accordance with syndrome differentiation principles | 20 min | 3 times/week × 4weeks |
| Li DD 2014(39) | Rome Ⅲ | electroacupuncture,  2/100 Hz dense–disperse wave; intensity 0.1–1.0 mA | ST36, PC6 | 30 min | 5 times/week × 4weeks |
| Ma CY 2014(41) | Rome Ⅲ | electroacupuncture,  2/100 Hz continuous wave | CV12, ST25, ST34 | 30 min | 5 times/week × 2weeks |
| Ma TT 2012(42) | Rome Ⅲ | electroacupuncture,  2/100 Hz frequency; intensity 0.5–1.5 mA | ST42, ST40, ST36, ST34 | 30 min | 5 times/week × 4weeks |
| Qiang LM 2018(43) | Rome Ⅲ | electroacupuncture,  2/100 Hz dense–disperse wave | ST36, SP6, SP4, PC6 | 30 min | 1 time/day × 30days |
| Sheng JW 2013 (44) | Rome Ⅲ | electroacupuncture,  25 Hz frequency; intensity 2–10 mA | ST36, PC6 | 30 min | 5 times/week × 4weeks |
| Tang KY 2023(45) | Rome Ⅳ | manual acupuncture | CV12, ST25, PC6, ST34, GB34, ST36, LR3 | 30 min | 1 or 3 times/week × 4weeks |
| Tu JF 2020(46) | Rome Ⅳ,  PDS | manual acupuncture | GV20, CV12, CV6, CV17, ST25, PC6, ST36, SP4 | 20 min | 3 times/week × 4weeks |
| Yang JW 2020(47) | Rome Ⅳ,  PDS | manual acupuncture | DU20, RN17, RN12, ST25, RN6, PC6, ST36, SP4 | 20 min | 3 times/week × 4weeks |
| Yu F 2020(48) | Rome Ⅳ | manual acupuncture | DU20, EX-HN1, DU24, DU28, GB13, PC6, LR3, RN12, ST36, SP4 | 30 min | 5 times/week × 4weeks |
| Zeng F 2012(50) | Rome Ⅲ,  PDS | electroacupuncture,  2/100 Hz frequency; intensity 0.1–1.0 mA | ST34, ST36, ST40, ST42 | 30 min | 5 times/week × 4weeks |
| Zheng H 2018(49) | Rome Ⅲ | electroacupuncture,  2/100 Hz intermittent wave; intensity 0.1–1.0 mA | ST36, PC6 | 30 min | 5 times/week × 4weeks |
| Zhou L 2019(51) | Rome Ⅳ | manual acupuncture | GV20, EX-HN1, PC6, CV12, ST36 | 30 min | 7 times/week × 4weeks |
| Jin YL 2015(52) | Rome Ⅲ | manual acupuncture | ST36, KI3 | 20-60 min | 3-4 times/week × 4weeks |
| Wang JJ 2015(53) | Rome Ⅲ,  PDS | manual acupuncture | ST36, PC6 | 30 min | 5 times/week × 4weeks |
| Yang ZQ 2011(54) | Rome Ⅲ | electroacupuncture,  2/100 Hz dense–disperse wave | CV12, ST36, PC6, ST25 | 30 min | 5 times/week × 4weeks |
| Yu SY 2010(55) | Rome Ⅲ | electroacupuncture,  2/100 Hz dense–disperse wave; intensity 0.1–1.0 mA | ST42, ST40, ST36, ST34 | 30 min | 5 times/week × 4weeks |

Abbreviations: PDS: postprandial distress syndrome
