# Supplementary figures and images for: Efficacy and safety of acupuncture for functional dyspepsia: an updated meta-analysis of randomized controlled trials
Source: Front Med (Lausanne). 2026 Feb 9;13:1718632. doi: 10.3389/fmed.2026.1718632 (PMC12926150; doi:10.3389/fmed.2026.1718632)

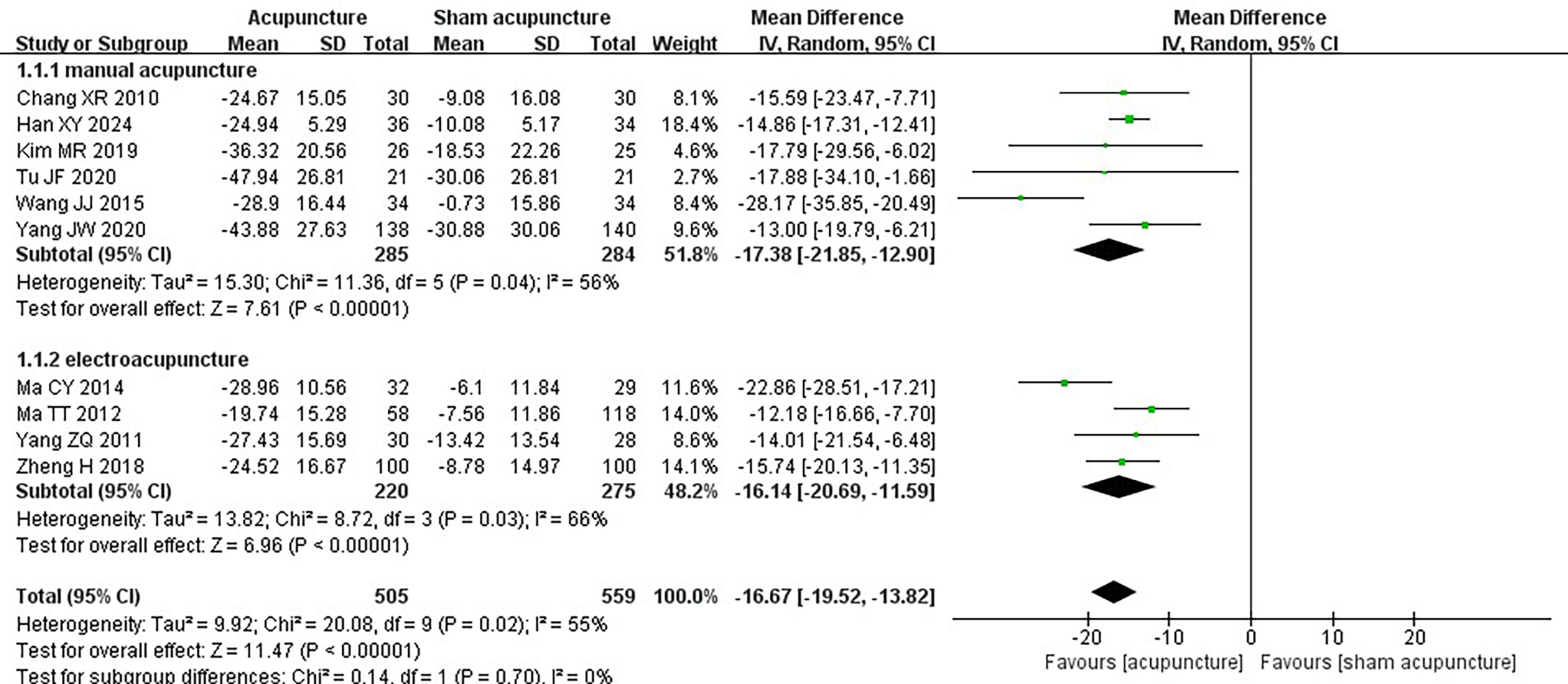

Supplement: Supplementary file 9 [file Image_1.jpeg]

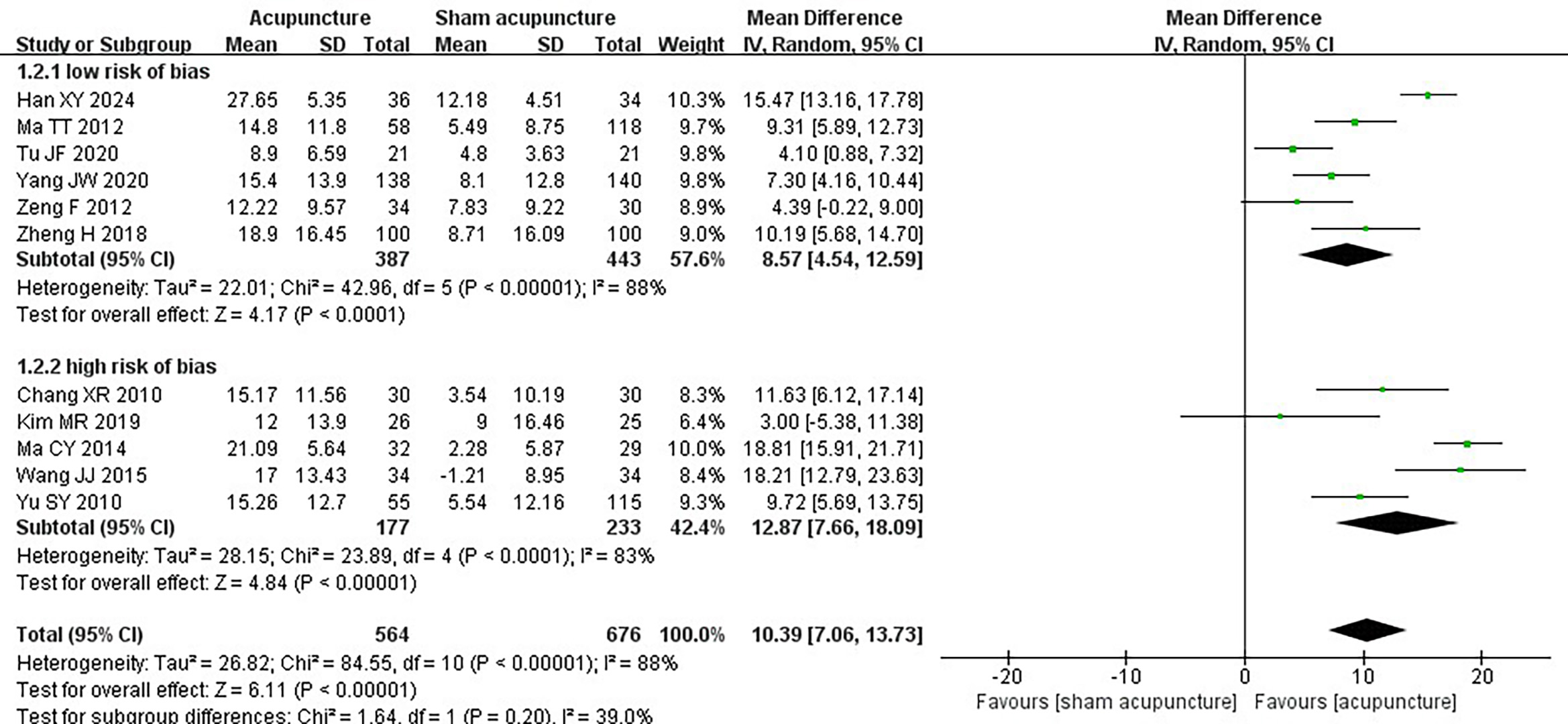

Supplement: Supplementary file 10 [file Image_2.jpeg]

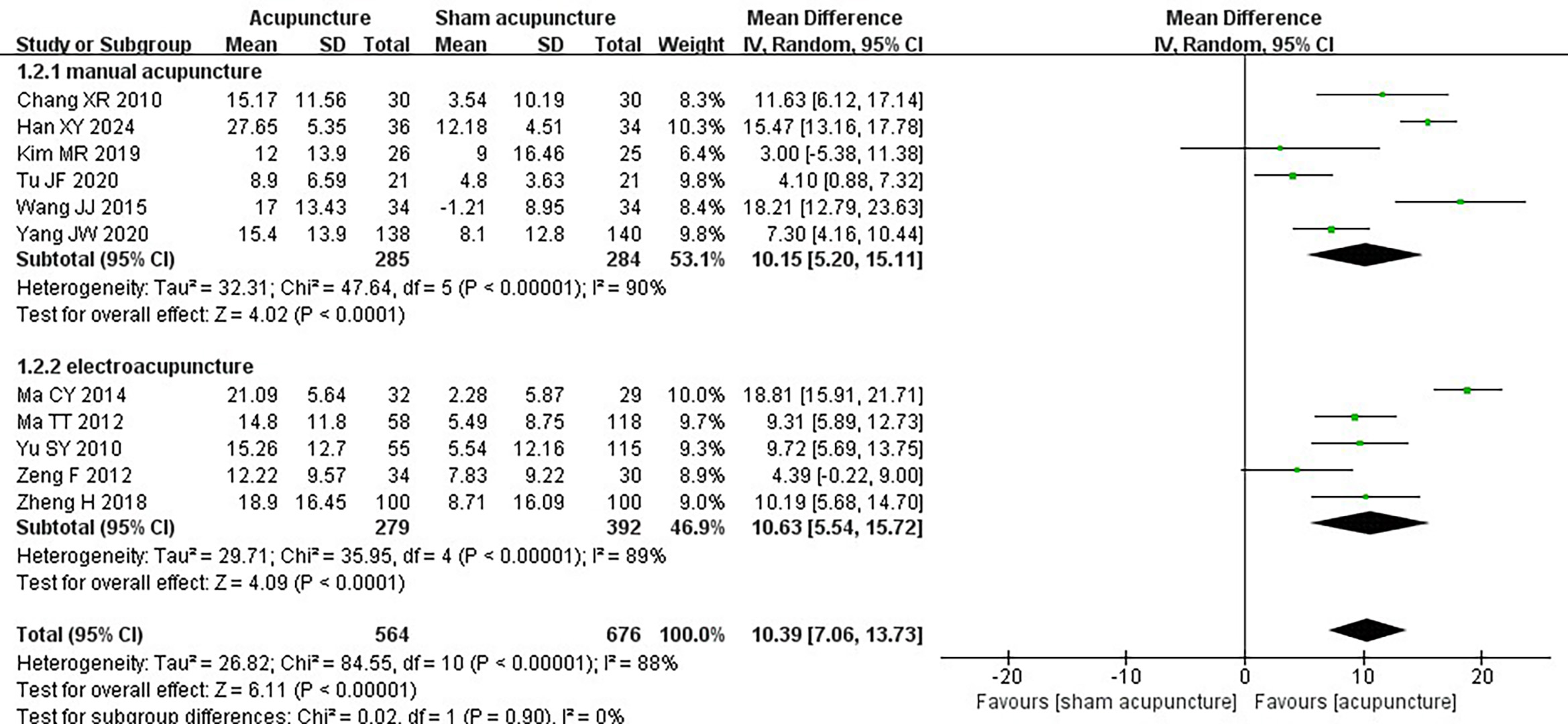

Supplement: Supplementary file 11 [file Image_3.jpeg]

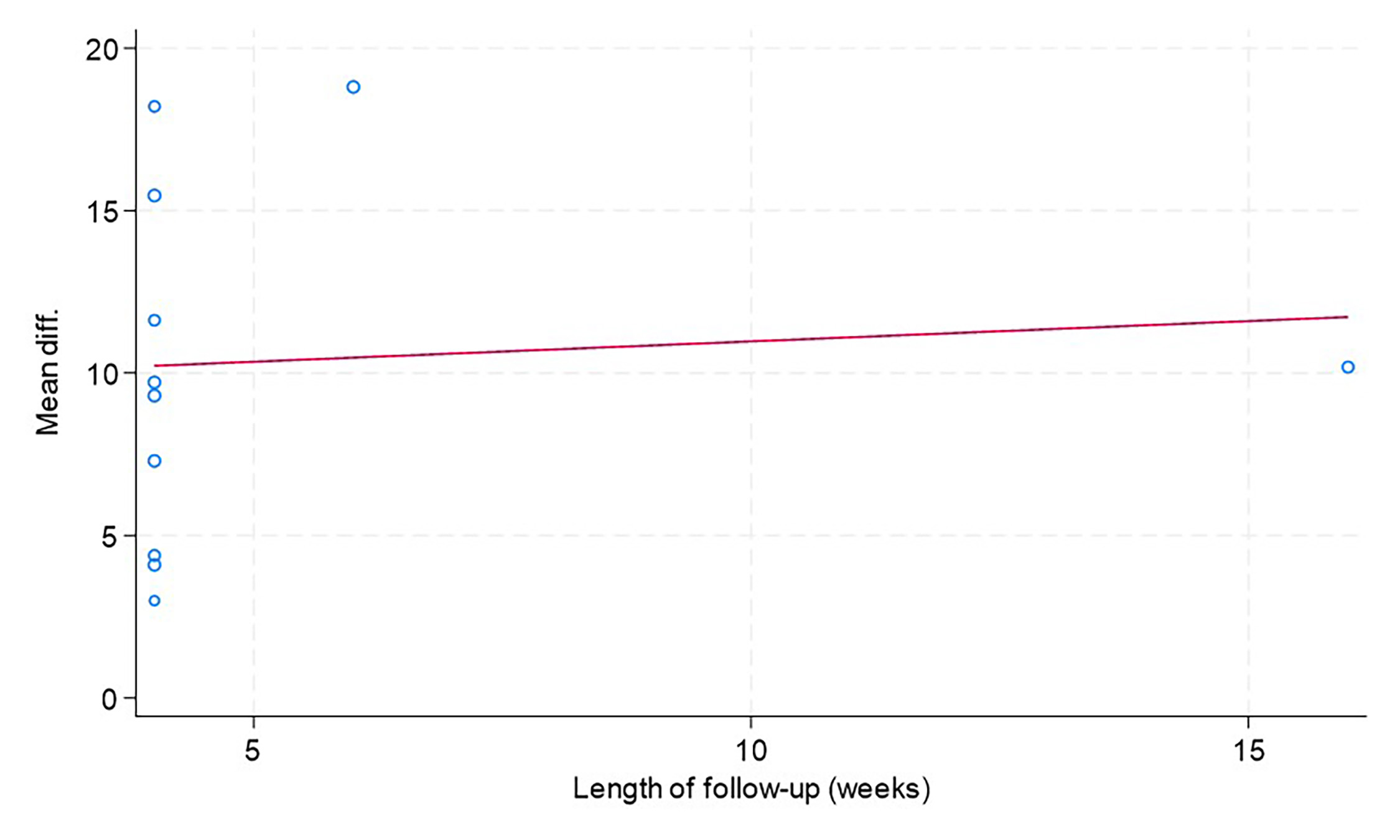

Supplement: Supplementary file 12 [file Image_4.jpeg]

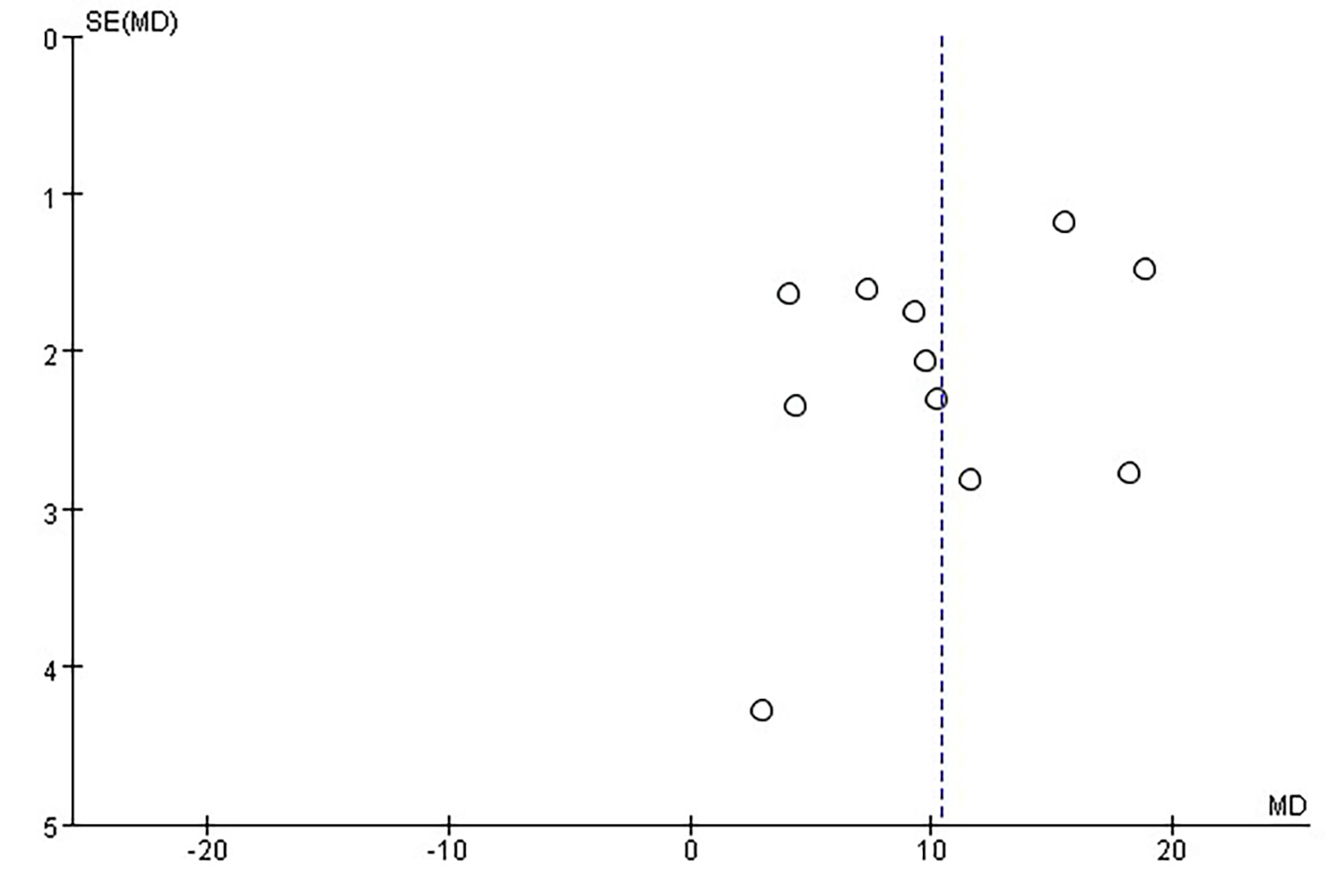

Supplement: Supplementary file 13 [file Image_5.jpeg]

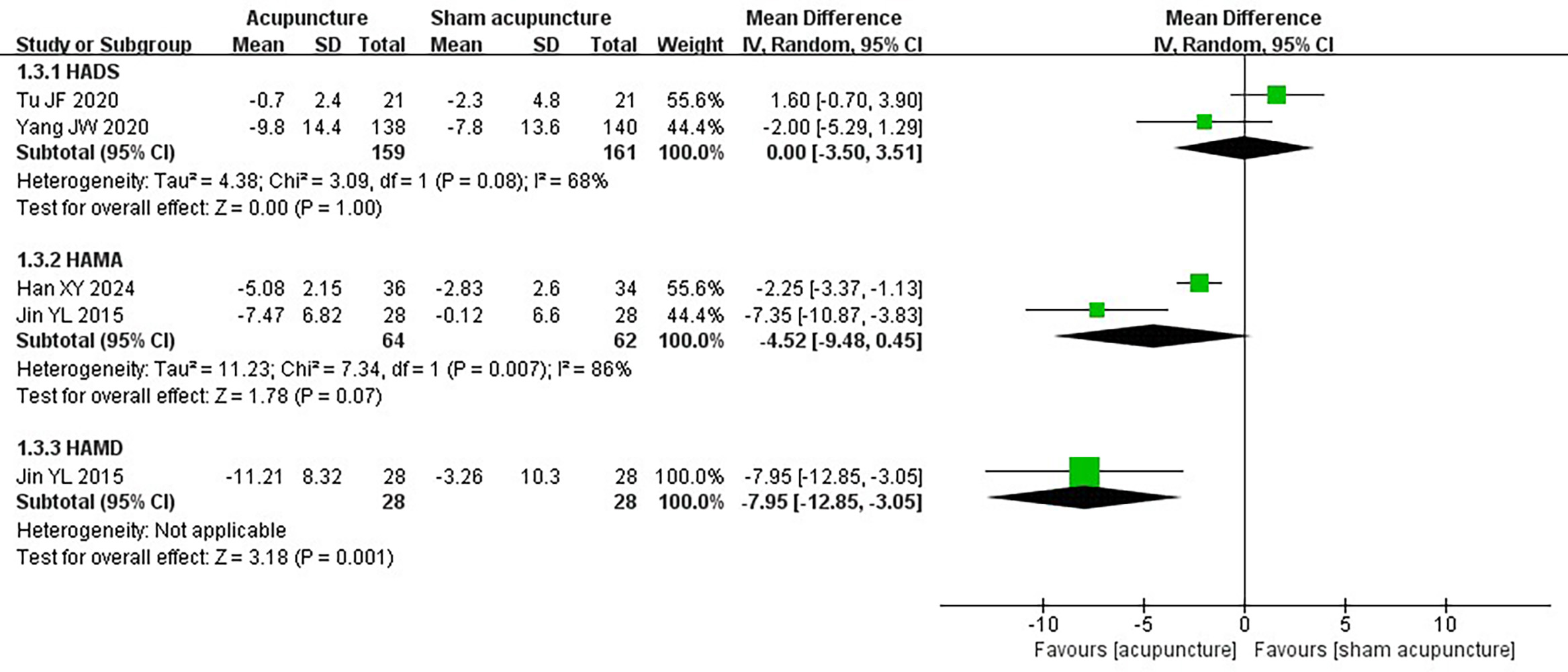

Supplement: Supplementary file 14 [file Image_6.jpeg]

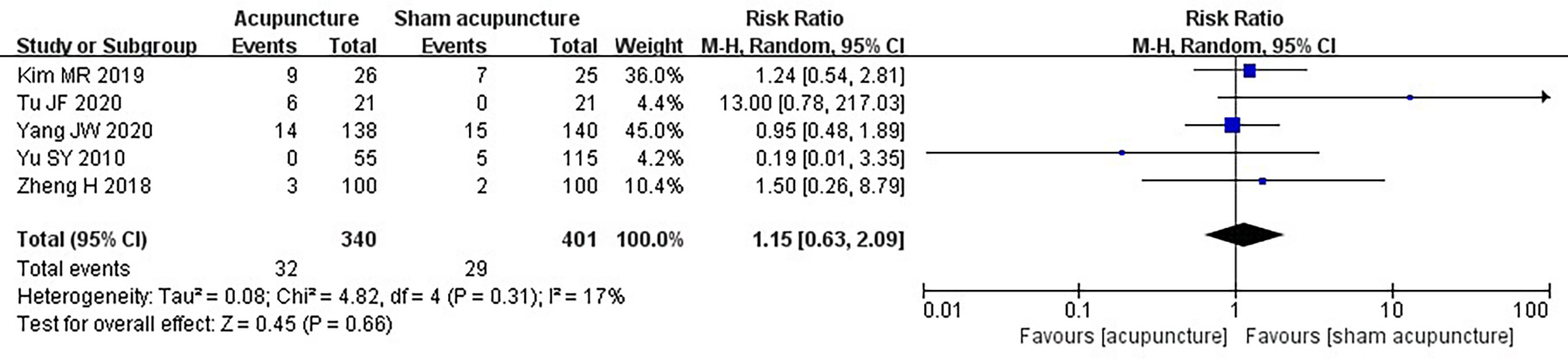

Supplement: Supplementary file 15 [file Image_7.jpeg]

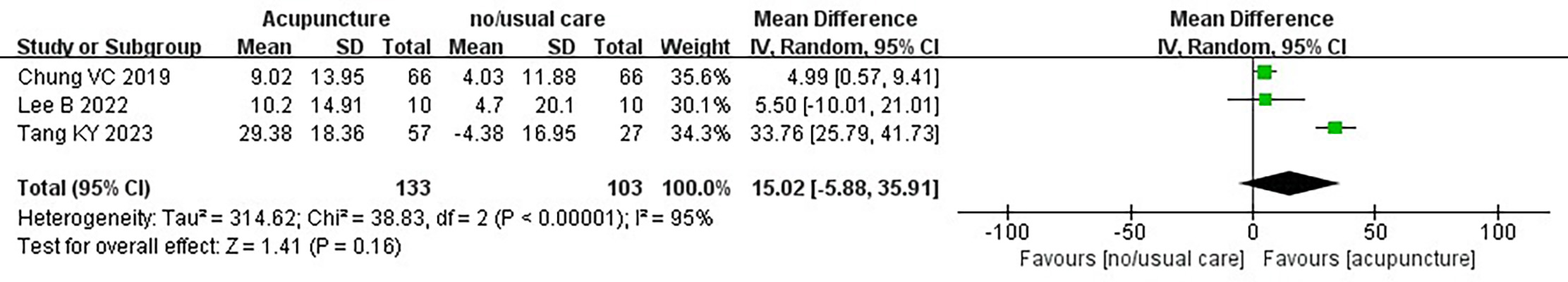

Supplement: Supplementary file 16 [file Image_8.jpeg]

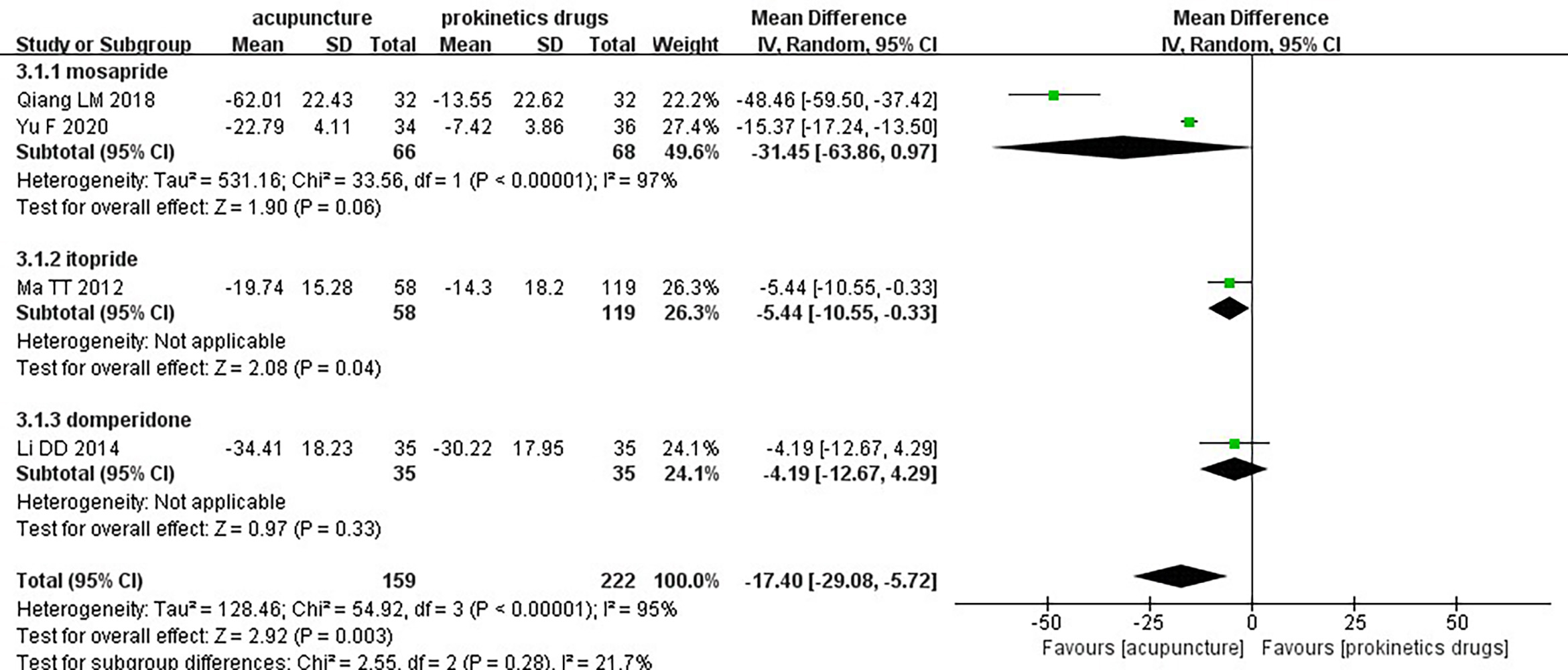

Supplement: Supplementary file 17 [file Image_9.jpeg]

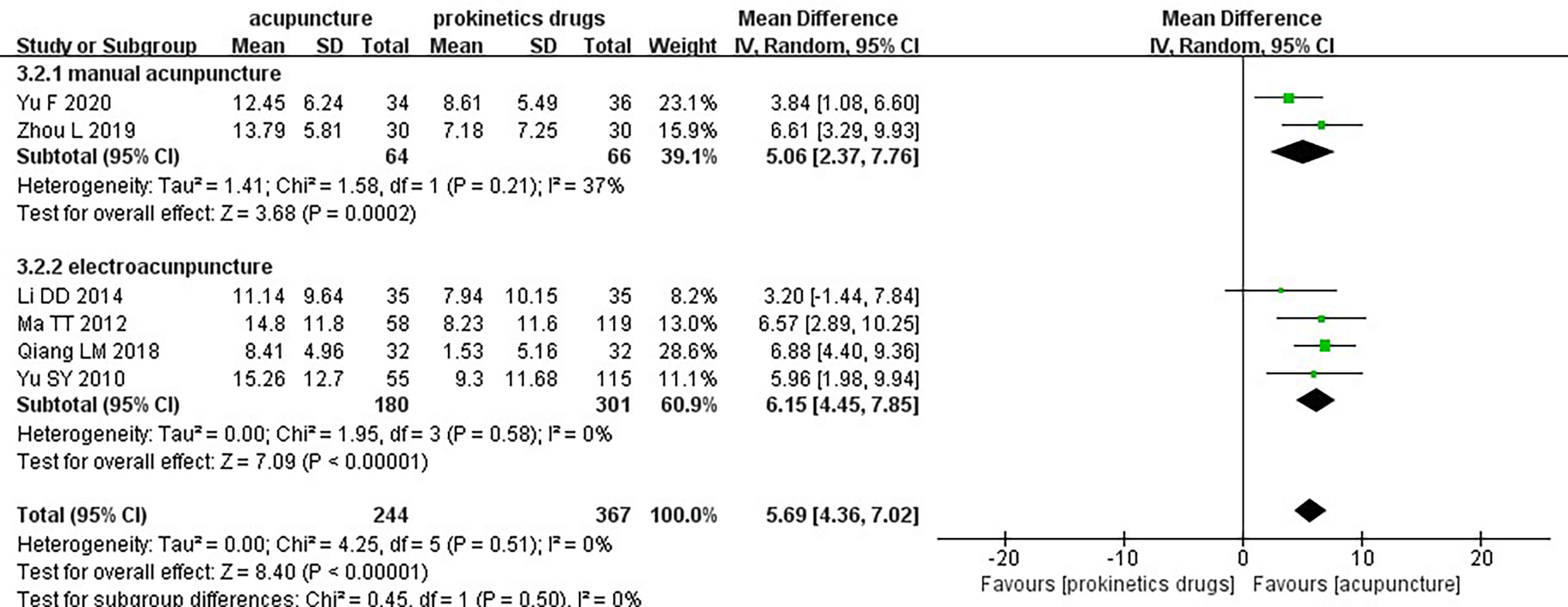

Supplement: Supplementary file 18 [file Image_10.jpeg]

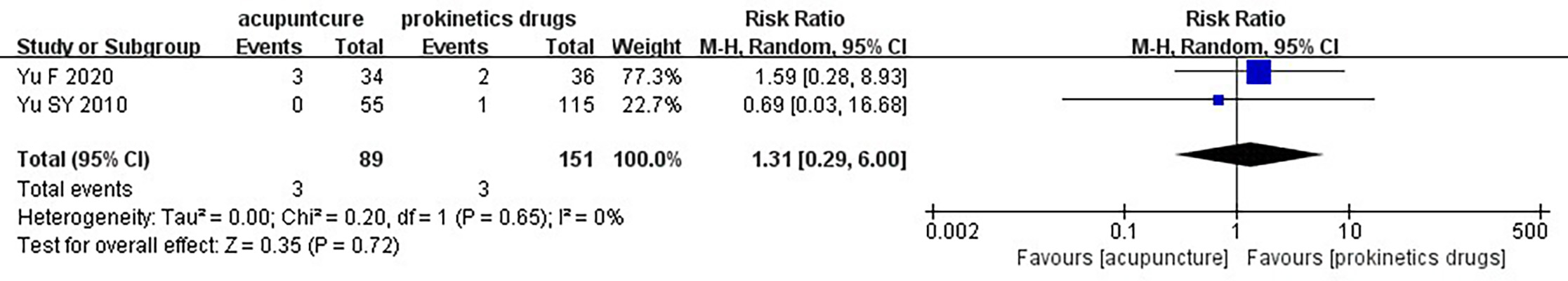

Supplement: Supplementary file 19 [file Image_11.jpeg]
